# 

**Published:** 1909-06

**Authors:** 


### The Library of the Bristol Medico-Chirurgical Society.

*The following donations have been received since the publication of the List in March:*

*May 31st*, 1909.

The Editor of *Albany Medical Annals* (1) .. .. 1 volume.
A. L. Flemming (2) .. .. .. .. .. 12 volumes.
R. G. Poole Lansdown, M.D. (3) .. .. .. 1 volume.
A. Shingleton Smith, M.D. (4) .. .. .. 1 volume.

Pamphlets have been presented by the Smithsonian Institution, the Director of the Public Health and Marine-Hospital Service of the United States, Mr. Paul Bush and Mr. L. M. Griffiths.

### SEVENTY-SECOND LIST OF BOOKS.

The titles of books mentioned in previous lists are not repeated.

The figures in brackets refer to the figures after the names of the donors, and show by whom the volumes are presented. The books to which no such figures are attached have either been bought from the Library Fund, or received through the *Journal*.

*Atlas of Clinical Medicine, Surgery and Pathology* (Edited by J. Hutchison) 2 vols. .. .. .. .. .. .. 1901-07
**Bateson, W.** .. *Genetics* .. .. .. .. .. .. .. .. .. .. .. .. (2) 1908
**Bennett, Sir W. H.** *Massage in Recent Fractures* .. .. 4th Ed. 1909
**Bramwell, B.** *Clinical Studies.* Vol. VI .. .. .. .. .. (4) 1908
**Browne, J.** .. [*Myographia nova*] (plates only) .. .. .. (3) 1684
**Childe, C. P.** .. *Operative Nursing and Technique* .. .. .. .. 1909
**Clayton, E. C.** *A Compendium of Food Microscopy* .. .. .. 1909
**Crichton-Browne, Sir J.** *Parcimony in Nutrition* .. .. .. .. 1909
*Encyclopedia and Dictionary of Medicine and Surgery*, *Green's* Vol. X .. .. .. .. .. .. .. .. .. .. 1909
**Fowler, J. S.** .. *Infant Feeding* .. .. .. .. .. .. .. .. .. .. 1909
**Glassington, C. W.** *Golden Rules of Dental Surgery* .. .. 3rd Ed. [N.D.]
**Gresswell, G. & A.** *Health, Morals and Longevity* .. .. .. .. 1909
**Hay, J.** .. *Graphic Methods in Heart Disease* .. .. .. .. 1909
*Heredity and Disease, Discussion on* .. .. .. .. .. .. .. .. .. 1909
**Hogarth, A. H.** *Medical Inspection of Schools* .. .. .. .. .. 1909
**Hopf, L.** .. *The Human Species* .. .. .. .. .. .. .. .. .. 1909
**Hort, E. C.** .. *Rational Immunisation in Pulmonary Tuberculosis* 1909
**Hunter, W.** .. *Severest Anæmias.* Vol. I .. .. .. .. .. .. 1909
**Husband, H. A.** *The Students' Pocket Prescriber* .. New Ed. 1909

**Hutchinson, Sir F. Treves and J.** *A Manual of Operative Surgery.* Vol. I .. .. .. .. .. .. .. .. 3rd Ed. 1909
**Lane, W. A.** .. *The Operative Treatment of Chronic Constipation* .. 1909
**Lithgow, R. A. D.** *Heredity* .. .. .. .. .. .. .. .. .. .. (2) 1889
**Lock, R. H.** .. *Variation, Heredity and Evolution* .. .. .. .. (2) 1907
**Macdonald, Isabel** *Home Nursing* .. .. .. .. .. .. .. .. .. 1909
**Minett, E. P.** .. *Differential Diagnosis of Bacteria* .. .. .. .. 1909
**Muir, R.** .. *Studies on Immunity* .. .. .. .. .. .. .. .. .. 1909
*New and Non-official Remedies* .. .. .. .. .. .. .. .. .. .. 1909
**Parkes, L. C.** .. *House Drainage in Relation to Health* .. .. .. 1909
**Patten, S. N.** .. *Heredity and Social Progress* .. .. .. .. (2) 1903
**Peake, G. A.** .. *Notes on Dental Anatomy* .. .. .. .. .. .. 1908
**Probyn-Williams, R. J.** *Golden Rules of Anæsthesia* .. 3rd Ed. [1908]
**Reid, G. A.** .. *The Principles of Heredity* .. 2nd Ed. (2) 1906
„ .. *The Present Evolution of Man* .. .. .. (2) 1896
**Rentoul, R. R.** *Proposed Sterilization of Degenerates* .. .. (2) 1903
*Research Defence Society, Publications of the* .. .. .. .. .. .. 1909
**Ribot, T.** .. *Heredity* .. .. .. .. .. .. .. 3rd Ed. (2) 1875
**Saleeby, C. W.** *Psychology* .. .. .. .. .. .. .. .. .. (2) [N.D.]
„ *Heredity* .. .. .. .. .. .. .. .. .. .. (2) [N.D.]
**Smith, E. A.** .. *Suture of Arteries* .. .. .. .. .. .. .. .. .. 1909
**Still, G. F.** .. *Common Disorders and Diseases of Childhood* .. .. 1909
**Thomson, J. A.** *Heredity.* .. .. .. .. .. .. .. .. .. .. (2) 1908
**Treves and J. Hutchinson, Sir F.** *A Manual of Operative Surgery.* Vol. I .. .. .. .. .. .. .. .. 3rd Ed. 1909
**Weismann, A.** *The Germ-Plasm.* (Tr. by W. N. Parker and H. Rönnfeldt) .. .. .. .. .. .. .. .. (2) 1893
**Whitelocke, R. H. A.** *Sprains and allied Injuries of Joints* .. .. 1909
**Wright, Sir A. E.** *Studies on Immunisation* .. .. .. .. .. .. 1909

### TRANSACTIONS, REPORTS, JOURNALS, etc.

Albany Medical Annals .. .. .. .. .. .. .. (1) Vol. XXIX 1908
American Journal of Ophthalmology, The .. .. .. Vol. XXV 1908
Archives of Pediatrics .. .. .. .. .. .. .. .. Vol. XXV 1908
Bookseller, The .. .. .. .. .. .. .. .. .. .. .. .. .. 1908
Boston Medical and Surgical Journal, The .. .. Vol. CLIX 1908
Bristol Medico-Chirurgical Journal, The .. .. Vol. XXVI 1908
British Journal of Children's Diseases, The .. .. .. Vol. V. 1908
British Journal of Dermatology, The .. .. .. .. Vol. XX 1908
British Journal of Tuberculosis, The .. .. .. .. Vol. II 1908
Bulletin de l'Académie de Médecine .. .. Tom. LVII, LVIII 1907
Clinical Journal, The .. .. .. .. .. .. .. Vol. XXXII 1908
College of Physicians of Philadelphia, Transactions of the Vol. XXX 1908
Écho médical du Nord, L' .. .. .. .. .. .. .. .. .. .. 1908
Gazette de Gynécologie .. .. .. .. .. Tom. XXII, XXIII 1907–08
Gazette des Hôpitaux .. .. .. .. .. .. .. .. .. .. .. .. 1908
Glasgow Medical Journal, The .. .. .. .. .. Vol. LXX 1908
Hospital and Charities, Burdett's .. .. .. .. .. .. .. .. 1909

| | | |
|---|---|---|
| Hospitals—Medical Reports of the Central London Throat and Ear Hospital .. .. .. .. .. .. .. | Vol. I | 1908 |
| Saint Bartholomew's Hospital Reports .. | Vol. XLIV | 1909 |
| Indian Medical Gazette, The .. .. .. .. .. .. | Vol. XLIII | 1908 |
| Johns Hopkins Hospital Bulletin, The .. .. .. | Vol. XIX | 1908 |
| Journal of Experimental Medicine, The .. .. .. .. | Vol. X | 1908 |
| Journal of Medical Research, The .. .. .. .. | Vol. XIX | 1908 |
| Journal of Mental Science, The .. .. .. .. .. | Vol. LIV. | 1908 |
| Journal of Nervous and Mental Disease, The .. | Vol. XXXV | 1908 |
| Journal of Obstetrics and Gynæcology of the British Empire | Vol. XIV | 1908 |
| Journal of Tropical Medicine, The .. .. .. .. | Vol. XI. | 1908 |
| Journal of the Royal Sanitary Institute .. .. | Vol. XXIX | 1909 |
| Library Association Record, The .. .. .. .. .. .. .. | Vol. X | 1908 |
| Library, The .. .. .. .. .. .. .. .. .. | N.S., Vol. IX | 1908 |
| Medical Annual, The .. .. .. .. .. .. .. .. .. .. .. | | 1909 |
| Medical Press and Circular, The .. | [Vols. CXXXVI, CXXXVII] | 1908 |
| Medico-Chirurgical Society of Edinburgh, The Transactions of the | Vol. XXVII | 1908 |
| Medico-Chirurgical Transactions .. .. .. .. .. | Vol. XC | 1907 |
| New York Obstetrical Society, Transactions of the .. .. .. .. | | 1908 |
| Nord médical, Le .. .. .. .. .. .. .. .. .. | Tom. XIV | 1908 |
| Ophthalmological Society, Transactions of the .. | Vol. XXVIII | 1908 |
| Revue de Thérapeutique .. .. .. .. .. .. .. .. .. | | 1908 |
| Revue génerale d'Ophtalmologie .. .. .. .. | Tom. XXVII | 1908 |
| St. Petersburger medicinische Wochenschrift .. .. .. .. .. | | 1908 |
| Smithsonian Report for 1907 .. .. .. .. .. .. .. .. .. .. | | 1908 |
| Studies from the Department of Pathology of the College of Physicians and Surgeons, Columbia University | Vol. XI | 1906–08 |
| Surgery, Gynecology and Obstetrics .. .. .. .. | Vol. VII | 1908 |
| West London Medical Journal .. .. .. .. .. | Vol. XIII | 1908 |
| Wiener klinische Wochenschrift .. .. .. .. .. .. .. .. | | 1908 |
| Year-Book of Scientific and Learned Societies .. .. .. .. .. | | 1908 |
| Zeitschrift für Urologie .. .. .. .. .. .. .. .. .. | Bd. II. | 1908 |
| Zentralblatt für Chirurgie .. .. .. .. .. .. .. .. .. .. | | 1908 |
| Zentralblatt für Gynäkologie .. .. .. .. .. .. .. .. .. | | 1908 |
| Zentralblatt für innere Medicin .. .. .. .. .. .. .. .. | | 1908 |

---

### MEETINGS OF SOCIETIES.

### Bristol Medico-Chirurgical Society.

### Local Medical Notes.

**University College, Bristol.**—Faculty of Medicine :—

EXAMINATION SUCCESSES.

F.R.C.S.Eng.—*Primary Examination:* A. J. M. Wright, M.R.C.S.Eng.

M.B., B.S.Lond.—*Final Examination:* E. R. Holborow. *Honours:* C. A. Joll, B.Sc., M.R.C.S., L.R.C.P., L.D.S. (*distinguished in Medicine, Pathology, Forensic Medicine and Hygiene, Surgery*), *University Medal.* Preliminary Science Examination—

*Experimental Physics:* G. M. Campbell. *Inorganic Chemistry:* Alice H. Christie, B. N. Walmsley. *Organic Chemistry:* F. W. T. Clemens.

Conjoint Board.—*Chemistry:* A. G. Morris. *Biology:* C. L. Emmerson, G. F. Fawn, J. W. Gilbert, R. B. Johnson, C. J. D. May. *Anatomy and Physiology:* A. G. T. Fisher. *Medicine:* J. F. H. Morgan, S. H. Kingston, H. H. S. Templeton, C. A. Joll,* F. C. Nicholls.* *Midwifery:* R. C. Clarke, A. J. Wigmore, H. R. B. Hull, C. A. Joll.* *Surgery:* C. A. Joll,* T. B. Dixon.*

M.R.C.S., L.R.C.P.—P. C. Field, H. C. Waldo.

L.D.S., R.C.S.Eng.—*Mechanical Dentistry:* J. D. Melhuish, A. H. S. Palmer, S. Saxton, R. A. Slade, B. W. Tyson. *Dental Metallurgy:* C. V. Osborne, A. H. S. Palmer, S. Saxton, R. A. Slade, B. W. Tyson. *Final Examination: Part I*—F. J. Hayman; *Part II*—R. J. Burton.*

### APPOINTMENTS.

Fells, Arthur, M.B., C.M.Edin.—Clinical Assistant at Bristol Eye Hospital.

Goulden, Charles, F.R.C.S.Eng.—Ophthalmic Surgeon, Oldham Infirmary.

Rossiter, Harold T., M.R.C.S., L.R.C.P.Lond.—House Physician to Addenbrooke's Hospital, Cambridge.

Rutherford, James M., M.B., C.M.Edin.—Senior Resident Physician to Brislington House, Bristol.

Smith, L. Shingleton, M.B., B.C.Cantab.—Medical Officer to the Workhouse of the Brecknock Union.

Squire, E. W., M.B., B.S.Lond.—House Surgeon to the *Dreadnought* Hospital.

Fortescue-Brickdale, J. M., M.A., M.D.Oxon.—Lecturer in Pharmacology in the University of Oxford.

Cross, F. Richardson, F.R.C.S.—Bradshaw Lecturer to the Royal College of Surgeons for 1909.

Bazalgette, S., M.R.C.S., L.R.C.P.Lond.—First Assistant Medical Officer at the Fishponds Asylum.

Phillips, John R. P., M.R.C.S., L.R.C.P.Lond.—Junior Medical Officer at the Fishponds Asylum.

Jones, J. Ellington, M.R.C.S., L.R.C.P.—Honorary Skiagraphist to Bristol Orthopædic Hospital.

* Qualified.

### BRISTOL.

**The Royal Hospital for Sick Children and Women, Bristol.**—The annual meeting of the subscribers of this Institution was held on March 29th, under the presidency of the Lord Mayor. The medical report stated that during 1908 the in-patients numbered 818 (751 children and 67 women), compared with 784 (727 children and 57 women) in 1907. 3,610 children and 1,541 women were treated in the out-patient department, as against 3,276 children and 1,456 women in the previous year. The financial statement was satisfactory, and showed that as a result of a special appeal for increased subscriptions, £5,386 had been raised. The committee has consequently been able to discharge the adverse balance of £3,998 remaining at the close of 1908, and also defray the cost and equipment of the cottage required for the accommodation of special scarlet fever cases, and the sum of £639 has been carried forward as a small nucleus of a fund for new buildings for the out-patient department.

**Bristol Eye Dispensary.**—The ninety-seventh annual meeting of the supporters of this Institution was held on April 6th. The report stated that the number of patients attending in 1908 had been 2,408, an increase of 53 over the preceding year. The total receipts were £207, and the total expenditure was £217. An increase in the number of regular subscribers was asked for. The examination of school children has meant an appreciable increase in the number of refraction cases, and there was a general feeling at the meeting that the education authorities should not allow this work to fall upon a charitable institution. In the evening the Dispensary Staff exhibited a number of interesting cases to medical men who were invited. There was a good muster, and this was felt to be a successful innovation.

**Fishponds Asylum.**—During 1908 the admissions numbered 247 (103 men and 144 women). The percentage of recoveries on the admissions were 42.42, and compared favourably with previous years; 84 patients were discharged as recovered. Deaths numbered 140 (74 males and 66 females), being 15.09 on the average of resident patients. Of 123 *post-mortems* death was due to natural causes. The highest number of patients resident at one time was 915.

**Hospital Sunday Fund at Bristol.**—Up to April 2nd the collections in aid of the Lord Mayor of Bristol's Hospital Sunday Fund were £1,853. Last year £1,859 was received, and as there are still some collections co come in, it is evident that the total of that year will be exceeded.

**University of Bristol.**—This title is now not a thing of the future, but an actual fact, as was announced on May 18th, when the Bill passed the Privy Council. The news was received in

Bristol with the ringing of church bells, and numerous flags were displayed to celebrate the event.

At a recent meeting of the Bristol City Council it was decided, with little opposition, to support the University by a rate of 1d. in the £, which will mean an addition of £7,000 per annum to the income of the University. The late Sir Frederick Wills left £5,000 towards the scheme.

It has been decided to form an Association of Alumni of the University, following the lead of the Universities of Liverpool and Toronto, where great advantages to the University have followed the formation of such associations.

Steps are being taken for the formation of a unit of the Officers' Training Corps amongst the staff and students of the University. The matter has been hanging fire, but now that a charter has been obtained things may soon take a definite shape, particularly as the Council are interesting themselves in the matter.

The Bristol Hairdressers' Association has made a grant of £20 for a research into the nature and means of prevention of sycosis barbæ. This, it is suggested, constitutes an example to other industries of the help they may expect from the University as a centre of scientific investigations.

### CARDIFF.

**Cardiff Medical School.**—Although it is not at present possible to take the complete medical curriculum at the Cardiff College, the number of students from the medical school who have already become qualified is 123. A great stimulus to the establishment of a Faculty of Medicine will no doubt be given by the recently issued report to the Treasury of the Committee appointed to inquire into certain matters connected with the University of Wales and the Welsh University Colleges. Sir Donald MacAlister, President of the General Medical Council, who was a member of the Committee, has appended a note to the report which has special reference to the Cardiff Medical School. He points out that the large international shipping trade of Cardiff makes it a natural centre for the study of tropical diseases, and he suggests that if a special school were organised in connection with a completed medical department of the Cardiff College, students and practitioners from other parts of the country would be attracted to it. He also expresses the opinion that Cardiff possesses all the requisites for a complete school of medicine, though he is careful to say that new buildings are an essential requisite, an assertion which no one will controvert.

